# Body adiposity dictates different mechanisms of increased coronary reactivity related to improved *in vivo* cardiac function

**DOI:** 10.1186/1475-2840-13-54

**Published:** 2014-02-27

**Authors:** Evangelia Mourmoura, Valérie Chaté, Karine Couturier, Brigitte Laillet, Guillaume Vial, Jean-Paul Rigaudiere, Béatrice Morio, Corinne Malpuech-Brugère, Kasra Azarnoush, Luc Demaison

**Affiliations:** 1 Université Joseph Fourier, Laboratoire de Bioénergétique Fondamentale et Appliquée, BP 53, Grenoble F-38041, France; 2 U1055 INSERM, Grenoble F-38041, France; 3 INRA, UMR 1019 Nutrition Humaine, CRNH Auvergne, Clermont-Ferrand, France; 4 Clermont Université, Université d’Auvergne, UMR 1019 Nutrition Humaine, Clermont-Ferrand, France; 5 INSERM UMR-1060, Laboratoire CarMeN, Université Lyon 1, INRA USC1362, INSA de Lyon, Facultés de médecine Rockefeller et Charles Merieux Lyon-Sud, Lyon F-69003, France; 6 Heart surgery Department, G. Montpied Hospital, Clermont-Ferrand University Hospital, Clermont-Ferrand, France

**Keywords:** Obesity, Cardiac function, Coronary reserve, Nitric oxide, Cyclooxygenase, Arachidonic acid

## Abstract

**Background:**

Saturated fatty acid-rich high fat (HF) diets trigger abdominal adiposity, insulin resistance, type 2 diabetes and cardiac dysfunction. This study was aimed at evaluating the effects of nascent obesity on the cardiac function of animals fed a high-fat diet and at analyzing the mechanisms by which these alterations occurred at the level of coronary reserve.

**Materials and methods:**

Rats were fed a control (C) or a HF diet containing high proportions of saturated fatty acids for 3 months. Thereafter, their cardiac function was evaluated *in vivo* using a pressure probe inserted into the cavity of the left ventricle. Their heart was isolated, perfused iso-volumetrically according to the Langendorff mode and the coronary reserve was evaluated by determining the endothelial-dependent (EDV) and endothelial-independent (EIV) vasodilatations in the absence and presence of endothelial nitric oxide synthase and cyclooxygenase inhibitors (L-NAME and indomethacin). The fatty acid composition of cardiac phospholipids was then evaluated.

**Results:**

Although all the HF-fed rats increased their abdominal adiposity, some of them did not gain body weight (HF- group) compared to the C group whereas other ones had a higher body weight (HF+). All HF rats displayed a higher *in vivo* cardiac activity associated with an increased EDV. In the HF- group, the improved EDV was due to an increase in the endothelial cell vasodilatation activity whereas in the HF+ group, the enhanced EDV resulted from an improved sensitivity of coronary smooth muscle cells to nitric oxide. Furthermore, in the HF- group the main pathway implicated in the EDV was the NOS pathway while in the HF+ group the COX pathway.

**Conclusions:**

Nascent obesity-induced improvement of cardiac function may be supported by an enhanced coronary reserve occurring via different mechanisms. These mechanisms implicate either the endothelial cells activity or the smooth muscle cells sensitivity depending on the body adiposity of the animals.

## Background

High-fat (HF) diets are now common in the Western societies and may induce insulin resistance (IR) when the absorbed lipids are rich in saturated fatty acids (SFAs) and poor in n-3 polyunsaturated fatty acids (PUFAs) [1]. IR, often associated with hyperglycemia, abdominal obesity, hypercholesterolemia, hypertriglyceridemia and hypertension, constitutes one of the major causes of the metabolic syndrome. This last pathology predisposes patients to numerous chronic diseases such as type 2 diabetes, atherosclerosis, myocardial infarction, stroke and renal disease [2,3].

Abdominal obesity could be the main catalyst of IR and associated detrimental events [4] and could have a potent effect on myocardial function. In the long term, this abnormality is known to depress cardiac function and induce severe cardiomyopathy [5]. The reduced cardiac function has already been observed *in vivo* and *in vitro* in several animal models of obesity including the Zucker Diabetic Fatty rat (ZDF) [6], post-natal over nutrition-related rat [7,8] and HF-fed rat [9]. However, as exemplified by post-natal over nutrition-induced obesity, the *in vivo* ejection fraction increases firstly until the third month of age and decreases only after a longer period of obesity [8]. The initial increase in cardiac output is a normal adaptation designed to compensate for the increased body weight and related energy expenditures. It could be due to the augmentation of blood and left ventricle telediastolic volumes encountered in those situations [8] which would contribute to increase myocardial contractility through the Frank Starling’s relationship. However, that improvement is only transitory and could be responsible for the later cardiomyopathy.

Associated with the improved cardiac output of the early phase of obesity an increased coronary flow and reserve could occur. Yet, all studies concerning this last parameter showed either an upholding [7,10-12] or a depression of the coronary reserve [13-18]. There is only one study performed on the isolated coronary arteriole [11] mentioning an obesity-related enhancement of the sensitivity of the coronary smooth muscle cells (SMCs) to nitric oxide (NO), which was however not associated with an improved endothelial-dependent vasodilatation (EDV).

The present study was aimed at evaluating the effects of the precocious development of obesity induced by a HF diet on the *in vivo* cardiac function and the *ex vivo* coronary reserve and at analyzing the mechanisms by which these alterations occurred. For that purpose, male Wistar rats were fed for 3 months with a diet high in saturated and monounsaturated fatty acids (MUFAs). All HF-fed animals gained abdominal adiposity compared to the control group, but only some of them gained body weight. This allowed the estimation of the effects of normal weight (gain in abdominal obesity, but no gain in body weight) and moderate (gain in abdominal adiposity and body weight) obesity on the cardiac mechanical function and coronary reserve. In order to explain the observed phenomena, the sensitivity to NO of SMCs (endothelial-independent vasodilatation or EIV) and endothelial cell vasodilatation activity (ECVA) were determined. The influence of nitric oxide synthase (NOS) and cyclooxygenase (COX) inhibitors on the EDV was also estimated. Furthermore, in order to investigate the molecular mechanism of each effect, the fatty acid composition of cardiac phospholipids was evaluated.

## Methods

### Ethical approval

All experiments followed the European Union recommendations concerning the care and use of laboratory animals for experimental and scientific purposes. All animal work was approved by the local board of ethics for animal experimentation (Cometh) and notified to the research animal facility of our laboratory (authorization n° 152_LBFA-U1055-LD-03).

### Experimental animals and diet

Ninety male Wistar rats from an inbred colony were housed two per cage in our animal facility at 3 months of age. Forty-five of them were randomly assigned to be maintained on standard carbohydrate (C) (16.1% proteins, 3.1% lipids, 60% cellulose; A04, Safe, France) diet and the fifty-one others were fed a high-fat (HF) (31.5% proteins, 54% lipids (50% lard, 4% soya-bean oil w/w), 7% cellulose) diet over a twelve-week period. The energy from fat in this diet typically represents more than 50% of total calories [19,20] as in an average Western diet. After analysis of the fatty acid composition of the diets chosen we found that the standard diet contained approximately 24% of SFAs, 23% of MUFAs, 48% of n-6 PUFAs and 4.5% of n-3 PUFAs while the HF diet contained 37% of SFAs, 46% of MUFAs, 15% of n-6 PUFAs and 1.2% of n-3 PUFAs. All groups were fed *ad libitum* with free access to water and their body weight and food intake were recorded twice weekly. It should be noted that the protein content of the HF diet was 2-fold higher because of the lower food intake (g/day) in these rats due to the higher energy density. This allowed a similar daily protein intake in all rat groups.

In a first set of experiments, eight rats fed with either the control or the HF diet were used to determine cardiac function in the *in vivo* situation.

In a second series of experiments, six rats were fed with the control diet and twelve animals with the HF diet. These animals were used to evaluate the effect of the diet on the body and blood compositions, *ex vivo* cardiac mechanical function and coronary reactivity, activities of the respiratory chain complexes and fatty acid composition of cardiac phospholipids. On the day of the experiment, the rats were weighed and heparinized (1,000 I.U./kg) via the saphenous vein before their sacrifice. The rats were allowed to eat up to the beginning of the experiment. Blood samples were collected for further biochemical analysis and their visceral (VAT) and peri-renal (PRAT) adipose tissue was weighed. Abdominal adipose tissue (AAT) weight was the sum of VAT and PRAT weights.

In a third set of experiments, twenty-four rats were fed with the control diet and thirty-two with HF one. The rats of each dietary group were further divided in four subgroups (n = 6 per group for the control rats and n = 4 for the HF-fed rats) in order to evaluate the effect of the diet on the *ex vivo* coronary reactivity in the absence of inhibitors, in the presence of L-NAME (a NOS inhibitor), in the presence of indomethacin (a COX inhibitor) and in the presence of both inhibitors.

### Oral glucose tolerance test (OGTT)

An OGTT was performed 2 weeks before the sacrifice. Food was removed from rats 18 h before they were given orally a glucose dose (1 g glucose/kg body weight, between 08.00 and 10.00 am). Blood samples were collected from the tail vein in heparinized tubes immediately before glucose administration to determine the basal glucose and insulin values and 5, 25, 40, 60 and 180 min after. Glucose values were determined with a glucose analyzer (ACCU-CHECK Active, Softclix). After centrifugation (3000 × g, 7 min, 4°C) plasma samples were stored at −20°C until insulin determination using a radioimmunoassay kit (SRI-13 K, Millipore, Molheim, France). The total area under the curve (AUC) for glucose was then calculated in order to evaluate the glucose tolerance as previously used by Cortez *et al*. [21].

### In vivo cardiac function

After deep anesthesia with sodium pentobarbital (40 mg/kg), the animal throat was dissected in order to isolate the right carotid artery. That artery was clamped at the proximal level in order to stop the blood flow arriving from the heart and a pressure gauge (Millar Instruments Inc., Houston, Texas) related to an amplifier (Gould) was introduced downstream through a small incision in the vessel. The clamp was removed and the gauge was progressively introduced in the aorta and then in the left ventricle cavity. The left ventricle pressure was then monitored after a 10 min-period of stabilization. The heart rate, systolic, diastolic, developed pressures, dP/dt max and dP/dt min were determined from the recordings.

### Ex vivo cardiac function

For the *ex vivo* Langendorff assessment of the cardiac function, a rapid thoracotomy was performed on the rats and the heart was immediately collected in Krebs-Heinselet solution maintained at 4°C. It was then rapidly (less than 1 minute from the chest opening to avoid problem of cellular damages and preconditioning) perfused at constant pressure according to the Langendorff mode with a Krebs–Heinselet buffer containing (in mM) NaCl 119, MgSO_4_ 1.2, KCl 4.8, NaHCO_3_ 25, KH_2_PO_4_ 1.2, CaCl_2_ 1.2 and glucose 11 mM as sole energy substrate. The buffer was maintained at 37°C and continuously oxygenated with carbogen (95% O_2_/5% CO_2_). A latex balloon connected to a pressure probe was inserted into the left ventricle and filled until the diastolic pressure reached a value of 7–8 mmHg. This allowed the monitoring of heart rate, systolic, diastolic and left ventricle developed pressures throughout the perfusion protocol. A pressure gauge inserted into the perfusion circuit just upstream the aortic cannula allowed the evaluation of the coronary pressure. The heart was perfused at constant pressure of 59 mmHg for 30 minutes and the coronary flow for each heart was evaluated by weight determination of 1-min collected samples at the 25^th^ min of perfusion. After this period, the heart was perfused at constant flow conditions, for which the flow rate was adjusted in order to obtain the same coronary flow as in the preparation at constant pressure. The systolic, diastolic and left ventricle developed pressures as well as the heart rates were determined after 10 min of perfusion at forced flow in order to allow a satisfying stabilization of the heart. The left ventricle developed pressure (LVDP) was calculated by substracting the diastolic pressure to the systolic pressure. The rate-pressure product (RPP) was defined as the product of left ventricle developed pressure and heart rate. All the parameters were recorded and analyzed with a computer using the HSE IsoHeart software (Hugo Sachs Elektronik, March-Hugstetten, Germany).

### Ex vivo coronary reactivity

After the evaluation of the cardiac function at constant flow, we assessed the effects of the HF diet on the coronary reactivity. After the 10-min equilibration period at constant flow, the coronary tone was raised by using the thromboxane analog U46619 (30nM), which was constantly infused into the perfusion system near the aortic cannula at a rate never exceeding 1.5% of the coronary flow. This allowed the obtainment of a coronary pressure between 90 and 110 mmHg. In our model of perfusion at forced flow, the aortic pressure equaled the coronary pressure and changes in the coronary tone triggered modifications of the aortic pressure. Changes in aortic perfusion pressure were thus used to monitor changes in coronary tone. Furthermore, this experimental model permitted the evaluation of the coronary microvasculature reactivity since in the rat the overall coronary pressure is determined mainly by the coronary resistance vessels. Relaxation responses to acetylcholine (Ach, 4, 10, 20, 40, 60, 80 and 100 pmoles) and sodium nitroprusside (SNP, 100, 200, 400, 600, 800 and 1000 pmoles) injections were determined reflecting the EDV and EIV respectively.

The choice of these ACh doses was made in order to avoid the ACh-induced negative inotropic effect, which decreases the EDV. This dose was never reached in our study, since the cardiac mechanical function was never impaired by the ACh injection. From the obtained dose response curves, it is obvious that the ACh-induced vasodilatation was saturated for the highest tested doses. Consequently, the maximal ACh-induced vasodilation was reached and the coronary reserve measured amongst the ACh doses used in our study.

The dilatation amplitude was calculated as the ratio between the maximal decrease in the coronary pressure and the coronary pressure just before the injection of the dilatation agents. Since the heart weight and coronary volume were subjected to intra- and inter-group variations, a correction was performed to normalise the input-function of the vasodilatation agents according to the coronary flow. The dose–response curve between the amount of vasodilatation agent injected and the maximal vasodilatation was then fitted to a logarithm function for each heart, which allowed the fulfillment of statistical analyses. Moreover, the vasodilatation activity of the endothelial cells (ECs) was also estimated from the corrected EDV and EIV curves. For each heart and each injected ACh dose, the amount of SNP (reflecting the amount of vasodilator agents) necessary to obtain the same percentage of ACh-induced vasodilatation was extracted from the EIV curve according to the formula: ECVA = e ^[(% ACh-induced dilatation - b) / a]^, where a and b are the coefficients of the theoretical EIV curve. The results were expressed in pmole equivalents of nitroprusside. At the end of the perfusion protocol, the hearts were freeze-clamped and stored at −80°C until the biochemical analyses were performed.

In another set of coronary reactivity experiments, the hearts of the control and HF groups were perfused as already described and the EDV was evaluated in presence of a NOS inhibitor (L-NAME 0.1 mM) or a COX inhibitor (indomethacin 2.5 μM). Finally, hearts were perfused in the simultaneous presence of L-NAME (0.1 mM) and indomethacin (2.5 μM).

### Enzymatic determinations

Activities of the respiratory chain complexes I, II, III and IV were determined as previously described [22]. Citrate synthase activity was evaluated according to Faloona and Sreere [23].

### Fatty acid composition of cardiac phospholipids

The phospholipid fatty acid composition was determined in cardiac homogenates as previously described [24]. The lipids were extracted according to Folch *et al*. [25]. The phospholipids were separated from non-phosphorus lipids using a Sep-pack cartridge [26]. After trans methylation, the fatty acid methyl esters were separated and analyzed by gas chromatography.

### Other biochemical determinations

All biochemical measurements (total cholesterol, triglycerides, glucose) were done in plasma samples by using an automated analyzer (HITACHI 912, Roche Diagnostics). Chemicals were obtained from Roche (Meylan, France). Proteins were measured using the bicinchoninic acid method with a commercially available kit (Thermo Scientific, Rockford, IL).

### Statistical analysis

Results are presented as mean ± S.E.M. Animal weight, metabolic parameters and data describing the cardiac mechanical and vascular function (left ventricular developed pressure, heart rate, rate pressure product, coronary pressure, and coronary flow) were contrasted across the two groups by one-way analysis of variance (ANOVA). Measures related to the action of the vasodilatation agents were treated with repeated-measures ANOVA to test the effect of the group (external factor), that of the amount of dilatation agent (internal factor) and their interactions. When required, group means were contrasted with a Fisher’s LSD test. A probability (p) less than 0.05 was considered significant. Statistical analysis was performed using the NCSS 2007 software.

## Results

### General data

During the experiments, analysis of the animal weight indicated that six rats of the HF-fed group did not gain weight compared to the control group during the 3-month dietary period, whereas the six others became significantly heavier than those of the control group. This delineated three distinct groups: i) the control (C) rats fed the control diet; ii) the animals whose weight did not differ from the control animals despite ingestion of the dense food (HF- group); and iii) the animals that gained weight due to the HF diet (HF+ group). However, the HF diet increased the adiposity of both HF rat groups. Table 1 shows statistical analyses indicating that the HF- group displayed a similar body weight as the C group and the HF+ group a higher body weight than the other two ones (+22 and +24% compared to the C and HF- groups, respectively, p < 0.05). The weight gain after this 3-month diet period was similar for the C and HF- groups (+21%) while it was significantly higher in the HF+ group (+24%). The cumulative dietary intake in the HF groups was 586 ± 15 and 644 ± 11 g/50 days/rat for the HF- and HF + groups respectively. The weight ratio between the PRAT and VAT was increased in the HF- group (2.0 ± 0.4 vs. 0.8 ± 0.1 in the C group, +150%, p < 0.05), but was intermediary in the HF + group (1.3 ± 0.4, not significant). Consequently, in the HF- rats, fat mass localized more in the peri-renal area and less in the visceral area compared to the HF + animals. The heart weight was decreased by the HF diet. However, that reduction was significant in those animals remaining lean (−18% for the HF- group, p < 0.05), but a tendency remained in the HF+ group.

**Table 1:** Animal characteristics

	**C**	**HF-**	**HF+**
Final body weight	438 ± 7^a^	429 ± 10^a^	534 ± 8^b^
Body weight gain	90 ± 3^a^	88 ± 4^a^	129 ± 6^b^
Heart weight	57 ± 2^a^	47 ± 3^b^	48 ± 6^a,b^
PRAT	1.2 ± 0.1^a^	3.6 ± 0.7^b^	3.2 ± 0.7^b^
VAT	1.5 ± 0.2^a^	1.9 ± 0.2^a,b^	2.5 ± 0.3^b^
AAT	2.7 ± 0.3^a^	5.4 ± 0.7^b^	5.8 ± 0.9^b^
Glucose	5.06 ± 0.34	5.17 ± 0.17	5.31 ± 0.05
Insulin	153 ± 17	106 ± 13	145 ± 26
Triglycerides	0.97 ± 0.06^a^	1.61 ± 0.25^b^	1.24 ± 0.04^a,b^
Total cholesterol	0.53 ± 0.01^a^	0.91 ± 0.07^b^	0.84 ± 0.06^b^
Total AUCglucose	29077 ± 1048^a^	32298 ± 1167^a,b^	34364 ± 2060^b^

The number of experiments was 6 per group. The body weight and body weight gain was expressed in g, the heart weight in mg of dry weight per 100 g of body weight, the weight of the perirenal (PRAT), visceral (VAT) and abdominal (AAT) adipose tissues in g of wet weight per 100 g of body weight, the concentration of glucose in mM, the concentrations of triglycerides and total cholesterol in g/l, the insulin concentration in mU/l and the total area under the curve (AUCglucose) in arbitrary units. C: control group; HF-: high fat-fed animals without weight gain compared to the control rats; HF+: high fat-fed animals with weight gain compared to the control rats; ^a, b^: two means located on a same line without a common letter are significantly different.

There were no statistically significant differences between the groups regarding the values of blood glucose and insulin on the day of sacrifice. However, the total area under the curve for glucose was moderately increased in the HF- group whereas it was significantly increased in the HF+ group (+18%, p < 0.05) compared to the control one. Total cholesterol concentrations were higher in the HF-fed rats (+72 and +58% for the HF- and HF+ compared to the control animals, p < 0.05). This was also the case for the triglyceride concentration in the HF- group (+66%, p < 0.05), but not in the HF+ group in which the difference did not reach significance (+28% only, not significant).

### In vivo cardiac function

Table 2 depicts the *in vivo* cardiac function in the HF diet-fed rats. The heart rate was not significantly affected by the diet. Conversely, the systolic pressure, LVDP, dP/dt max and dP/dt min were increased in the HF- group (+28, +33, +38% and + 36% compared to the C group respectively, p < 0.05), suggesting an augmented contractile function. These parameters were also moderately increased in the HF+ group (+17, +19, +24% and + 22% compared to the C group respectively, not significant).

**Table 2:** ***In vivo *** **cardiac function**

	**C**	**HF-**	**HF+**
HR	347 ± 20	397 ± 9	370 ± 24
SP	128 ± 4^a^	164 ± 14^b^	150 ± 14^a,b^
DP	21 ± 1	20 ± 1	21 ± 1
LVDP	108 ± 4^a^	144 ± 14^b^	129 ± 14^a,b^
dP/dt max	3350 ± 206^a^	4608 ± 530^b^	4163 ± 531^a,b^
dP/dt min	−3275 ± 180^a^	−4467 ± 457^b^	−3981 ± 513^a,b^

The number of experiments was 8, 4 and 4 for the C, HF- and HF + groups. The heart rate, pressures (systolic, diastolic and left ventricular developed pressures), dP/dt max and dP/dt min were expressed in beats/min, mmHg and mmHg/s. C: control; HF-: high fat-fed rats without weight gain; HF+: high fat-fed rats with weight gain; HR: heart rate; SP: systolic pressure; DP: diastolic pressure; LVDP: left ventricular developed pressure; dP/dt max: maximum rate of pressure change; dP/dt min: maximum rate of pressure change; ^a, b^: two means located on a same line without a common letter are significantly different.

### Ex vivo cardiac function

*The heart* rate was not affected by the diet (Table 3), but the LVDP was decreased in the HF+ group (−32% compared to the C group, p < 0.05). However, the reduction of LVDP was not significant in the HF- group. The RPP was always reduced by the HF diet irrespective of the body weight change of the animals (−27 and −28% for HF- and HF+ groups compared to the C group, p < 0.05). The decreased mechanical function occurred despite maintained coronary flows and pressures. Infusing the vasoconstrictor agent U46619 increased the coronary pressure to values ranging from 94 to 120 mmHg, but did not modify the rate pressure product (data not shown).

**Table 3:** ***Ex vivo *** **cardiac function**

	**C**	**HF-**	**HF+**
Heart rate	288 ± 29	248 ± 20	295 ± 20
LVDP	82 ± 10^a^	67 ± 2^a,b^	56 ± 7^b^
RPP	22.9 ± 1.5^a^	16.8 ± 1.7^b^	16.4 ± 1.7^b^
Coronary flow	42.9 ± 7.5	40.2 ± 6.7	42.9 ± 13.7
CP before U46619	62.8 ± 6.4	67.5 ± 5.9	68.4 ± 3.8
CP after U46619	94.0 ± 3.9	106.0 ± 8.5	120.4 ± 7.7

The number of experiments was 6 per group. The heart rate, pressures (left ventricular developed pressure or LVDP, coronary pressure or CP before and after U46619 infusion), rate pressure product (RPP) and coronary flow were expressed in beats/min, mmHg, mHg/min and ml/min/g of dry weight. C: control group; HF-: high fat-fed animal without weight gain compared to the control group; HF+: high fat-fed animals with weight gain compared to the control group; ^a, b^: two means located on a same line without a common letter are significantly different.

### Ex vivo coronary reactivity: effect of body weight change

**Figure 1:**
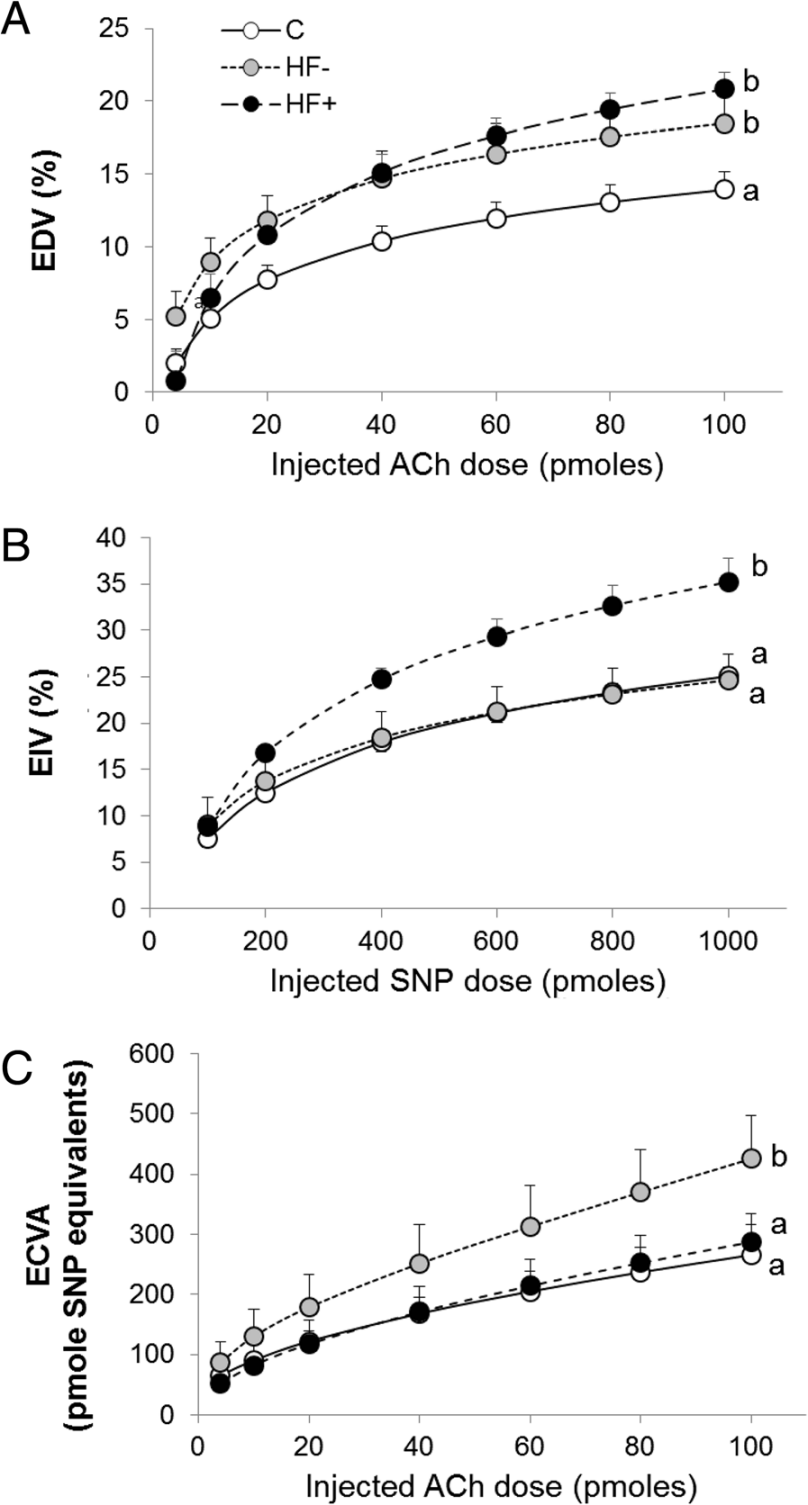
**Effects of the diets on the reactivity of the coronary microvasculature. (A)** Endothelial-dependent vasodilatation (EDV). **(B)** Endothelial-independent vasodilatation (EIV). **(C)** Endothelial cell vasodilatation activity (ECVA). C: control; HF- : HF-fed rats with no body weight change; HF+: HF-fed rats with body weight gain; Ach: acetylcholine; SNP: sodium nitroprusside. The number of experiments was 6 per group. a, b: significantly different.

The Figure 1A demonstrating the ACh-induced vasodilatation clearly indicates that the HF-diet increased that parameter. This was true for the HF- group (+36% when the administrated ACh dose was 60 pmoles, p < 0.05), but also for the HF+ group (+48% at 60 pmoles of administrated ACh, p < 0.05). As indicated by Figure 1B and 1C, the HF diet-induced increase in EDV did not originate from the same mechanism in the two HF-fed rat groups. When the HF-fed animals did not gain weight (HF- group), the increase in ACh-induced vasodilatation was due to an augmentation of the ECVA (Figure 1C). Indeed, the ECVA was increased in that group (i.e. +53% at 60 pmoles of administrated ACh, p < 0.05), whereas in the HF + group it displayed similar values as in the C group. On the contrary, when the HF-fed animals gained weight (HF + group), the increase in EDV was mainly due to an augmentation of the SNP-induced vasodilatation (Figure 1B), outlining the essential role of SMCs relaxation. In that last group, the EIV was increased by 39% at the administrated SNP dose of 600 pmoles compared to the C group (p < 0.05), whereas it remained similar to the one of the C group in the HF- animals.

### Ex vivo coronary reactivity: effects of NOS and cyclooxygenase inhibitors

The high coronary pressure induced by the U46619 infusion allowed the determination of the effects of the vasodilators ACh and SNP. The effects of each inhibitor were also calculated at 60 pm of the Ach injected dose.

**Figure 2:**
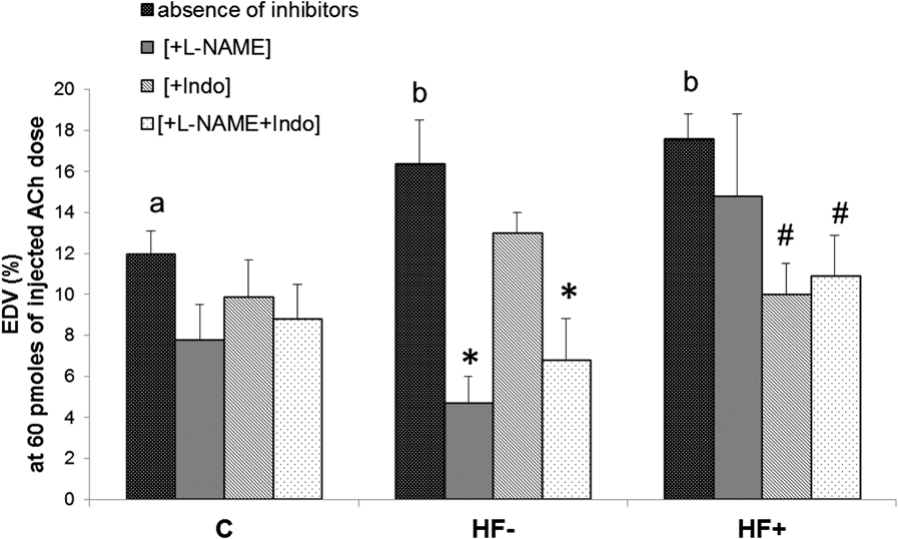
**Effects of HF diet, L-NAME and Indomethacin on the EDV of the coronary microvasculature at 60 pm of injected Ach dose.** The number of experiments was 6 for the control groups and 4 for the HF groups. C: control animals; HF-: HF-fed rats with no body weight change; HF+: HF-fed rats with body weight gain; Ach: acetylcholine. a, b: values without a common letter are significantly different; *: significant difference in the HF- group in the absence or presence of inhibitors; #: significant difference in the HF+ group in the absence or presence of inhibitors.

In the absence of inhibitor, the EDV was increased by the HF diet (i.e. +41% compared to the C group at the dose of 60 pmoles, p < 0.05, Figure 2). The presence of L-NAME reduced the EDV significantly only in the HF- group (−71% at 60 pmoles of injected ACh, p < 0.05) whereas indomethacin reduced this parameter only in the HF + group (−43% at 60 pmoles of injected ACh, p < 0.05). The presence of both inhibitors in the perfusate significantly reduced the EDV in both HF groups (−58% and −38% for the HF- and HF + groups respectively at 60 pmoles of injected ACh, p < 0.05).

### Respiratory chain complexes activities

Citrate synthase activity (Table 4) was of similar magnitude in the three groups. It was also true for C1 and C4 activities. C2 activity was decreased by the HF diet irrespective of the animal body weight change (−15% for the HF- and HF + groups compared to the C group when the values were expressed in unit per unit of citrate synthase, p < 0.05). That slight difference was however erased when the values were normalized to the amount of cardiac proteins. Finally, C3 activity appeared the most affected by the dietary intervention. That parameter was reduced by the HF diet when the animals did not gain weight (−26 and −28% for the values expressed in relation to the amount of mitochondria and the amount of myocardial proteins, p < 0.05), but was similar to the C group value for the HF+ group.

**Table 4:** Activities of the respiratory chain complexes in myocardial biopsies

**Enzyme**		**C**	**HF-**	**HF+**
CS	mU./mg	4.3 ± 0.1	4.4 ± 0.2	4.6 ± 0.4
C1	U./U. CS	0.24 ± 0.01	0.20 ± 0.02	0.21 ± 0.03
	mU./mg	1.05 ± 0.05	0.82 ± 0.07	0.99 ± 0.22
C2	U./U. CS	0.20 ± 0.01^a^	0.17 ± 0.01^b^	0.17 ± 0.01^b^
	mU./mg	0.85 ± 0.03	0.78 ± 0.05	0.79 ± 0.08
C3	U./U. CS	0.068 ± 0.004^a^	0.050 ± 0.005^b^	0.064 ± 0.002^a^
	mU./mg	0.29 ± 0.01^a^	0.21 ± 0.02^b^	0.29 ± 0.03^a^
C4	U./U. CS	0.024 ± 0.001	0.020 ± 0.002	0.020 ± 0.002
	mU./mg	0.101 ± 0.006	0.079 ± 0.006	0.094 ± 0.013

The number of experiments was 6 per group. Citrate synthase (CS) activities were expressed in mU./mg of myocardial proteins. Complex 1, 2, 3 and 4 (C1, C2, C3, C4) activities were expressed either in mU./mg of myocardial proteins (mU./mg) or in units/unit of citrate synthase activity (U./U. CS). C: control group; HF-: high fat-fed animals without weight gain compared to the control group; HF+: high fat-fed animals with weight gain compared to the control group; ^a, b^: two means located on a same line without a common letter are significantly different.

### Fatty acid composition of cardiac phospholipids

The fatty acid composition of myocardial phospholipids is presented in Table 5. SFAs were increased by the HF diet (+12 and +11% in the HF- and HF+ groups, p < 0.05) at the expense of the MUFAs (−20 and −26%, p < 0.05). However, the proportions of total PUFAs, n-6 PUFAs and n-3 PUFAs were not altered.

**Table 5:** Fatty acid composition of cardiac phospholipids

**Fatty acid**	**C**	**HF-**	**HF+**
C14:0	0.06 ± 0.01	0.08 ± 0.01	0.08 ± 0.01
DMA C16:0	3.22 ± 0.17	3.04 ± 0.17	3.34 ± 0.19
C16:0	13.15 ± 0.14	10.30 ± 0.48	11.38 ± 1.15
DMA C18:0	1.07 ± 0.05^a^	2.49 ± 0.12^b^	2.47 ± 0.20^b^
C18:0	21.27 ± 0.09^a^	27.42 ± 0.26^b^	25.86 ± 0.71^c^
C22:0	0.04 ± 0.01^a^	0.06 ± 0.01^b^	0.07 ± 0.01^b^
C24:0	0.15 ± 0.01^a^	0.06 ± 0.01^b^	0.05 ± 0.01^b^
SFA	38.82 ± 0.25^a^	43.44 ± 0.85^b^	43.24 ± 1.81^b^
C16:1 n-7	0.51 ± 0.05^a^	0.12 ± 0.01^b^	0.12 ± 0.05^b^
C16:1 n-9	nd	0.05 ± 0.03	0.07 ± 0.02
C18:1 n-7	5.24 ± 0.19^a^	2.91 ± 0.05^b^	2.60 ± 0.35^b^
C18:1 n-9	3.24 ± 0.04^a^	4.02 ± 0.07^b^	3.72 ± 0.27^a,b^
C20:1 n-9	0.07 ± 0.01	0.07 ± 0.01	0.07 ± 0.01
MUFA	8.91 ± 0.24^a^	7.17 ± 0.09^b^	6.57 ± 0.51^b^
C18:2 n-6	26.49 ± 1.27^a^	18.65 ± 0.49^b^	22.82 ± 1.08^c^
C18:3 n-6	0.02 ± 0.01^a^	0.01 ± 0.01^b^	0.01 ± 0.01^b^
C20:2 n-6	0.10 ± 0.01^a^	0.22 ± 0.01^b^	0.21 ± 0.02^b^
C20:3 n-6	0.28 ± 0.02^a^	0.59 ± 0.03^b^	0.64 ± 0.06^b^
C20:4 n-6	17.02 ± 0.34^a^	20.95 ± 0.84^b^	18.89 ± 1.10^a,b^
C22:4 n-6	0.24 ± 0.01^a^	0.50 ± 0.04^b^	0.46 ± 0.05^b^
C22:5 n-6	0.21 ± 0.03^a^	0.52 ± 0.03^b^	0.43 ± 0.05^b^
n-6 PUFA	44.36 ± 1.05	41.42 ± 0.48	43.45 ± 1.02
C18:3 n-3	0.02 ± 0.01	0.04 ± 0.01	0.05 ± 0.02
C20:5 n-3	0.12 ± 0.01	0.12 ± 0.03	0.09 ± 0.04
C22:5 n-3	0.75 ± 0.09^a^	1.82 ± 0.14^b^	1.81 ± 0.23^b^
C22:6 n-3	5.54 ± 0.22	6.00 ± 0.44	4.78 ± 0.46
n-3 PUFA	6.37 ± 0.21	7.98 ± 0.58	6.73 ± 0.67
Total PUFA	52.43 ± 0.12	49.40 ± 0.86	50.18 ± 1.55
n-6/n-3	7.00 ± 0.10^a^	5.28 ± 0.38^b^	6.62 ± 0.57^a^
EPA/AA	0.0068 ± 0.0008	0.0056 ± 0.0012	0.0043 ± 0.0019
EPA + DHA	5.66 ± 0.21	6.12 ± 0.44	4.87 ± 0.50
C18:0/C16:0	1.62 ± 0.02^a^	2.68 ± 0.11^b^	2.33 ± 0.18^b^
C18:1 n-7/C16:1 n-7	10.9 ± 1.6^a^	24.8 ± 0.9^b^	18.1 ± 5.6^a,b^
C20:2 n-6/C18:2 n-6	3.9 ± 0.1^a^	11.6 ± 0.8^b^	9.0 ± 0.7^c^
C22:4 n-6/C20:4 n-6	1.4 ± 0.1^a^	2.4 ± 0.2^b^	2.4 ± 0.1^b^
C22:5 n-3/C20:5 n-3	7.2 ± 1.7^a^	18.1 ± 5.2^a,b^	32.6 ± 9.3^b^
C16:1 n-7/C16:0	4.0 ± 0.6^a^	1.1 ± 0.1^b^	1.0 ± 0.3^b^
C18:3 n-6/C18:2 n-6	7.8 ± 0.5^a^	4.1 ± 1.4^b^	3.4 ± 1.1^b^
C20:4 n-6/C20:3 n-6	62.7 ± 5.6^a^	36.1 ± 2.8^b^	30.8 ± 4.7^b^

The number of experiments was 6 per group. Except for the different ratios, the results are expressed in percent of total fatty acids. The C22:4 n-6/C20:4 n-6 and C16:1 n-7/C16:0 ratios must be multiplied by 10^−2^. The C20:2 n-6/C18:2 n-6 and C18:3 n-6/C18:2 n-6 ratios must be multiplied by 10^−3^ and 10^−4^, respectively. DMA: dimethylacetal; SFA: saturated fatty acids; MUFA: monounsaturated fatty acids; PUFA: polyunsaturated fatty acids; EPA: eicosapentaenoic acid; AA: arachidonic acid; DHA: docosahexaenoic acid. C: control group; HF-: high fat-fed animals without weight gain compared to the control rats; HF+: high fat-fed animals with weight gain compared to the control rats; ^a, b, c^: two means located on a same line without a common letter are significantly different.

Some alterations occurred inside the lipid classes. The SFAs DMA C18:0, C18:0 and C22:0 were increased by the HF diet (+132, +25 and +63% compared to the C group, p < 0.05). Interestingly, the proportion of C18:0 was more increased in the HF- group (+29%, p < 0.05) than in the HF+ group (+22%, p < 0.05). In the MUFAs, the proportions of C18:1 n-9 was amplified by the HF diet at the detriment of the n-7 fatty acids (−76 and −47% for the C16:1 n-7 and C18:1 n-7, p < 0.05). In the n-6 PUFAs, the proportion of C18:2 n-6 was drastically decreased by the HF diet, but that reduction was ampler in the HF- group (−30%, p < 0.05) than in the HF+ group (−14%, p < 0.05). Except for the C18:3 n-6 which was also reduced, all the other n-6 PUFAs (C20:2 n-6, C20:3 n-6, C20:4 n-6, C22:4 n-6 and C22:5 n-6) were increased by the HF diet in a similar way for both HF- and HF + groups. The only exception was the C20:4 n-6, which was highly augmented in the HF- group (+23%, p < 0.05) and only moderately in the HF+ group (+11%, not significant). The HF diet-induced tendency to augment the carbon chain length of the membrane PUFAs was also true for the n-3 PUFAs. Indeed, the HF diet increased the proportion of C22:5 n-3 (+143 and +141% for the HF- and HF+ groups, p < 0.05).

As indicated by the C18:0/C16:0, C18:1 n-7/C16:1 n-7, C20:2 n-6/C18:2 n-6, C22:4 n-6/C20:4 n-6 and C22:5 n-3/C20:5 n-3 ratios, activities of the elongases were increased by the HF diet. Conversely, activities of the Δ9-, Δ6- and Δ5-desaturases were reduced as suggested by the reduction of the C16:1 n-7/C16:0, C18:3 n-6/C18:2 n-6 and C20:4 n-6/C20:3 n-6 ratios. Finally, the n-6 to n-3 PUFA ratio of cardiac phospholipids was decreased in the HF- group (−25%, p < 0.05), but not in the HF+ group.

## Discussion

The present study was aimed at determining the effects of a 3-month HF diet rich in SFAs and MUFAs on the cardiac function and at investigating the underlying mechanism at the level of coronary circulation. Despite its high percentage in fat content, the high-fat diet chosen for this study did not induce severe obesity in the animals but increased significantly their adiposity, which was accompanied or not by body weight gain. The increased adiposity of the animals was related to changes observed in the *in vivo* and *ex vivo* cardiac function as well as in the *ex vivo* coronary reactivity. However, the body weight change and thus the adipose tissue distribution seemed to affect the mechanism through which the changes at the coronary level occurred.

Feeding rats for 3 months with a HF diet containing large amounts of SFAs and MUFAs was expected to generally increase the body weight of the animals. During the experiments two distinct groups were observed in the HF-fed animals with one of them being characterized by no body weight change compared to the control group (HF-) and the other showing an increased body weight (HF+). These two groups displayed a similar increase in abdominal adiposity and were thus clearly different from the C group. The HF + group was composed of obese animals, since their body weight and abdominal adiposity were increased. However, the body weight was moderately increased compared to the C group. These animals were thus considered as moderately obese. The HF- rats were not distinctly obese, since they had an increased abdominal adiposity but no augmentation of body weight. As the case with high body weight and abdominal adiposity can occur in humans, the situation with low body weight and high abdominal adiposity also exists and refers to abnormal and hazardous body composition with high risk of chronic pathological events [27]. Indeed, this situation is interesting to study since it has been recently recognized in the literature the existence of different subtypes of obesity such as metabolically healthy but obese (elevated body fat but normal metabolic profiles) and metabolically obese, normal weight individuals that may be or not at increased cardiovascular risk. Furthermore, a new syndrome has been described lately in humans, the normal weight obesity syndrome, which is defined as a normal body mass index associated with increased body fat [28]. Thus, the study of these two HF subgroups allowed the study of two different subtypes of obesity that correspond to different situations of obesity occurring in humans.

The fact that the fat content of the HF diet chosen was not consisted entirely by saturated fat or that the protein content was doubled in our HF diet might explain the state of obesity that we found in our rats. It could also explain any differences concerning basic characteristics of the animals from previous studies, such as body weight and glucose levels [11,29]. Futhermore, Buettner *et al*. have shown that PUFA- or medium-chain fatty acids-rich diets did not induce insulin resistance in rats after a 12 week period [30]. Indeed, in the HF diet chosen for this study one can find not only SFA but also a percentage of PUFAs that could explain the metabolic results of the rats. This diet affected only the triglycerides and cholesterol levels of the rats indicating the beginning of a dyslipidemia. Furthermore, it provoked the development of glucose intolerance in the HF+ rats, which is consistent with previous studies [31]. Thus, the high-fat diet altered the metabolic profile of the HF-fed rats according to the percentage of the adiposity in their bodies with the HF- rats having a less obese profile compared to the HF+ rats.

The reason why the HF- animals did not gain body weight is not known, since we did not evaluate the food intake and the energy expenditure in our study. For that reason, we analyzed a previous study in which we determined the food intake. In that last study, the C diet was given to five rats and the HF diet to ten rats for a period of 50 days. As in the present study, the HF diet-fed animals were divided into two groups of equal sample size (n = 5) with the lightest and heaviest animals. The animal weight at the beginning of the experiment was similar in the three groups (321 ± 3, 318 ± 6 and 316 ± 3 g for the C, HF- and HF + groups, not significant). At the end of the fifty day-diet, the animal weight was significantly higher in the HF + group (445 ± 8 g) compared to the two other groups (411 ± 5 and 406 ± 8 g for the C and HF- groups, respectively). This perfectly fits with the results of the present study indicating that the HF-fed rats can either take weight compared to the control group or not. Indeed, the weight gain of the animals during the 3 month-feeding period was higher in the HF+ group compared to the two other ones. Interestingly, the weight gain paralleled that of the cumulative dietary intake in the HF groups, suggesting that the difference in weight gain was due to a difference in food intake. The palatability of the HF diet could thus be responsible for the observed differences. It could be high enough for the subgroup of rats becoming obese and insufficient for the other ones. However, the reduction of the n-6 to n-3 PUFA ratio of cardiac phospholipids observed in the HF- group could also limit the food intake. In the present study, despite the different body weight gains, the abdominal fat mass was of similar magnitude in the two subgroups of HF diet-fed rats, suggesting that the excessive caloric intake observed in the HF+ group was used to build up other tissues in the body. Another explanation for the lack of body weight gain in the HF- group could be an insufficient intake of proteins and a low lean mass build-up [32-35]. In the present study, the protein content of the HF diet was planned to be twice as high as that of the C diet in order to compensate for the lower food intake due to the dietary lipid enrichment. This could be not enough for certain animals in order to build up a sufficient amount of muscle proteins. Moreover, a reduced respiratory chain complex 3 activity which paralleled the decreased n-6 to n-3 PUFA ratio of membrane phospholipids was observed in the hearts of the HF- animals. This could lead to a lower rate of ATP production. If the energy available for biochemical synthesis was also reduced in the skeletal muscle, this could lead to decreased protein build-up and muscle mass formation. The phenomenon would not occur in the HF+ group, since the respiratory chain complex 3 activity and n-6 to n-3 PUFA ratio of membrane phospholipids were as high as in the C group. However, the formation of these two HF subgroups reveals that a HF diet can have differential effects on the body and blood composition of the individual.

The HF diet chosen for this study triggered an increase in the *in vivo* contractile function of the animals especially that of the HF- rats, whereas the HF+ rats had an intermediate profile between control and HF- rats. However, these results were not found in the *ex vivo* situation. Indeed, the *ex vivo* cardiac mechanical function was reduced by the HF diet, following the elevated adiposity of the animals irrespective of their body weight. That observation has already been presented in the literature after a HF diet period [9,31], after weight gain through post-natal overfeeding in the mouse [7] and in the rat [8] as well as in the ZDF rat [6]. This depressed *ex vivo* cardiac mechanical activity observed in this study could be related to changes in the cardiac metabolism related to the whole body glucose intolerance, the increased degree of saturation of the cardiac membranes as shown by the increase in the SFAs at the detriment of MUFAs [36] and the pro-inflammatory environment as indicated by the low ratio EPA/AA that predisposes to a balance of eicosanoids favoring platelet aggregation and inflammatory signaling [37,38]. However, an increased cardiac output is expected to occur with nascent low- and moderate-severity obesity [8]. Indeed, our *in vivo* cardiac function measurements suggest an augmented inotropism after the 3-month HF diet intake as already shown by measurements of the *in vivo* ejection fraction after post-natal overfeeding. That parameter is firstly increased at the age of 3 months before being reduced from the age of 5 months [8]. Thus, nascent obesity may lead to an increased cardiac output resulting from an increased cardiac mechanical function.

The further study of these two subgroups revealed the same profile of the *ex vivo* cardiac and coronary function after the HF diet but the results were related to different mechanisms at the level of coronary vessels depending on the body composition of the animals. These results indicate that the high-fat diet has an important effect on the adiposity of the individual, but not necessarily on the body weight, and that these changes in the adiposity are related to changes occurring at the level of cardiovascular function.

We then evaluated the *ex vivo* coronary reactivity of the animals according to Langendorff mode. We evaluated the global cardiac reactivity through estimation of changes in the aortic pressure, which in our model of Langendorff perfusion at fixed flow reflected mainly the pressure of the coronary micro vessels. The conductance vessels may also contribute to the aortic pressure, but no spasm and no atheroma plaque was expected to occur in our experimental conditions. This study reports for the first time that a 3 month HF diet triggered an increase in EDV of the coronary microvasculature. HF diet- or post-natal overfeeding-induced obesity has been associated with either a reduced [14-16,18] or a maintained [7,10,12] EDV of the coronary vessels. It has also been reported that glucose intolerance due to high-fat feeding does not alter myocardial perfusion during hyperemia [31]. However, Jerebolvszki *et al.*[11] reported a HF diet-induced increase in the sensitivity of pressurized coronary arterioles to NO, suggesting that the coronary reactivity can be increased in certain circumstances. This increase in the EDV of the HF-fed animals could augment the coronary reserve explaining the results of the *in vivo* situation.

The HF diet-induced inotropic effect that encountered *in vivo* in our experiments fits perfectly with the increased coronary reserve reported *ex vivo*. This mechanism could also explain results from previous studies reporting maintenance of myocardial perfusion or preserved contractile function after high-fat feeding [31,39,40]. Hence, early obesity triggers an *in vivo* increase in contractile function which is supported by an augmentation of the coronary reserve. The discrepancies between the *ex vivo* and *in vivo* situations observed in our study could be due to an increased left end diastolic volume reported to occur in overfed rats [8], which would stimulate the cardiac contractile function through the Frank Starling’s law in the *in vivo* situation.

The augmented EDV observed in our study paralleled the increase in abdominal fat mass, but was not related to an augmentation of body weight. It is possible that the increased fat mass at the abdominal level or at the pericardiac level if it also occurred acted on the coronary vessels through a change in adipocytokine release. Systemic leptin is increased with augmented adiposity [41] while adiponectin is reduced [42]. The resulting *in vivo* adiposity-related changes in coronary function could be retained *ex vivo* and contribute to the adaptation of myocardial function in nascent obesity. Indeed, it has been shown that obesity necessitates higher cardiac mechanical activity [43-45] due to augmented whole body energy expenditure. As already indicated, in our model of cardiac perfusion, we measured mainly the reactivity of the coronary microvasculature which determines myocardial perfusion. The increased coronary EDV observed in our study could reflect an augmented *in vivo* coronary perfusion due to an obesity-related increase in cardiac output.

Previous studies suggest that obesity may reduce NO levels mostly through increased oxidative stress [46] and that when NO bioavailability is reduced a compensatory mechanism takes place in order to maintain a normal coronary function [47]. Adaptation of coronary vessels is particularly important, as in the coronary circulation oxygen extraction is near maximal and any mismatch between blood supply and metabolic demand would deteriorate myocardial contractile function [48]. Furthermore, the increase in body mass, either muscular or adipose, requires higher cardiac output and expanded intravascular volume to meet the elevated metabolic requirements [45]. Thus, the vascular alterations observed in our study could help the coronary microvasculature to adjust the organ perfusion during physiological processes such as exercise. Otherwise the heart would not be able to respond to increased metabolic demands and lead eventually to ischemic incidents.

In order to evaluate the contribution of the main vasodilator pathways in the observed EDV, inhibitors that block NO production and COX were used during the perfusion protocol. The main results were the following: i) L-NAME reduced EDV in the HF- group, indicating the implication of NOS pathway in the enhanced ACh response in the HF group; ii) indomethacin decreased EDV in the HF+ group, implying an altered balance between COX-derived vasodilators and vasoconstrictors in the HF group. The HF diet seems to reduce the availability of vasoconstrictor mediators and maintain or even enhance that of vasodilators contributing eventually to the enhanced EDV. The analysis of the fatty acid content of the cardiac phospholipids also revealed that the arachidonic acid (AA, C20:4n-6) was increased in the HF- rat hearts which could lead eventually to an increase in the COX-vasoactive agents [49]; iii) association of L-NAME and indomethacin decreased the EDV in both HF- and HF+ groups. Thus, both NOS and COX pathways seem to be implicated in the HF-induced ACh response.

The study of the two HF subgroups (HF-, HF+) during the last set of experiments helped to elucidate the involvement of the NOS and COX pathways in the increased ACh-response of the HF rats. The augmented ACh- response of the HF- rats was due to an increase in the activity of endothelial cells, as shown by the ECVA diagram while that of the HF+ rats was due to an increased sensitivity of the smooth muscle cells to NO, as shown by the response to SNP injections. The mechanism explaining the increased EDV observed in the HF+ rats has already been described in the literature [11] and was explained by an increased sensitivity to NO of the SMC guanylate cyclase with consequent augmented cyclic guanosine monophosphate (cGMP) production and SMC relaxation. This fits well our results since L-NAME did not affect the EDV of the HF+ rats, indicating that NOS pathway was not affected, but since SMCs are more sensitive to NO which leads finally to increased EDV. Furthermore, the implication of COX-derived vasodilators seem to participate in the increased EDV of the HF+ rats as shown by the results of the indomethacin experiments. However, more original was the mechanism explaining the increased EDV observed in the HF- group. Indeed, these rats with normal weight obesity displayed an improved EDV which was strictly due to an increased ECVA, which was probably due to increased NOS signaling as shown by the results of the L-NAME experiments. Since the content of AA of myocardial phospholipids was increased in that group and not in the HF+ group, we also suspected the involvement of COX products in order to explain the increased ECVA. Indeed, indomethacin decreased the EDV of the HF-rats but not significantly, indicating that the NOS pathway remains the prominent pathway for the vasodilatation together with the activity of the endothelial cells. This relationship between NOS/COX pathways and endothelial/smooth muscle cells in the EDV seems to be reversed in the HF+ group, with the COX pathway having the most important role and the smooth muscle cells becoming more sensitive. Thus, the adipose tissue distribution seems to affect the mechanism through which the increased ACh response occurs in the HF-fed rats.

## Conclusions

In summary, our results showed that HF contribute to an augmentation of coronary EDV, which supports an improved cardiac mechanical activity necessary for the higher whole body energy expenditure of the individuals. Since the HF diet-fed rats did not display necessarily an increased body weight but had systematically a higher abdominal adiposity, the improved EDV was rather related to the increased abdominal fat mass. Despite all these alterations, the coronary microvasculature of HF fed obese rats adapts resulting to enhanced ACh responses in order to maintain an adequate tissue perfusion in cases of physiological processes of enhanced metabolic demand such as exercise. One of the most interesting points of our study is that the mechanisms of the coronary reactivity responsible for the improvement of the cardiac function depend on the percentage of body adiposity. Indeed, the augmented coronary reactivity of the HF rats was due either to an augmented sensitivity of the SMCs to NO in the rats displaying body weight gain compared to the control ones or to an improved ECVA in the animals with no body weight gain. To our knowledge, the last effect was never described in the literature and our results indicate that this was related to an enrichment of the myocardial membranes in AA. The increased arachidonic acid proportion resulted from a stimulation of elongases and inhibition of desaturases and was responsible for an increased production of cyclooxygenase end-product(s) with vasodilatation properties. Whatever the mechanism through which it occurs, the HF diet-induced increase in coronary reserve seem to favor the cardiac mechanical activity and thus the upholding of tissue perfusion and welfare of obese individuals.

## Abbreviations

AAT: Abdominal adipose tissue; Ach: Acetylcholine; ANOVA: Analysis of variance; ATP: Adenosine triphosphate; C: Control; C1: C2, C3 and C4, respiratory chain complexes 1, 2, 3 and 4; C16:1 n-7: Palmitoleic acid; C18:0: Stearic acid; C18:1 n-7: Cis-vaccenic acid; C18:1 n-9: Oleic acid; C18:2 n-6: Linoleic acid; C20:2 n-6: 11,14-eicosadienoic acid; C20:3 n-6: Dihomo-gamma-linolenic acid; C20:4 n-6 or AA: Arachidonic acid; C22:0: Behenic acid; C22:4 n-6: Adrenic acid; C22:5 n-6: Docosapentaenoic acid; C22:5 n-3: Clupanodonic acid; CaCl2: Calcium chlorure; CO2: Carbon dioxide; COX: Cyclooxygenase; DMA: Dimethylacetal; dP/dt max: Rate of left ventricle pressure rise; dP/dt min: Rate of left ventricle pressure decrease; ECs: Endothelial cells; ECVA: Endothelial cell vasodilatation activity; EDV: Endothelial-dependent vasodilation; EIV: Endothelial-independent vasodilatation; HF: High fat; HF-: High fat diet-fed rats with increased abdominal adiposity and maintained body weight; HF+: High fat diet-fed rats with increased abdominal adiposity and body weight; IR: Insulin resistance; KCl: Potassium chlorure; KH2PO4: Potassium dihydrogenophosphate; L-NAME: L-nitro-arginine-methyl-ester; LSD: Least significant difference; LVDP: Left ventricular developed pressure; MgSO4: Magnesium sulfate; MUFAs: Monounsaturated fatty acids; NaCl: Sodium chlorure; NaHCO3: Sodium hydrogenocarbonate; NO: Nitric oxide; NOS: Nitric oxide synthase; OGTT: Oral glucose tolerance test; O2: Dioxygen; P: Probability; PRAT: Peri-renal adipose tissue; PUFAs: Polyunsaturated fatty acids; RPP: Rate pressure product; SFAs: Saturated fatty acids; SMCs: Smooth muscle cells; SNP: Sodium nitroprusside; VAT: Visceral adipose tissue; ZDF: Zucker diabetic fatty.

## Competing interests

The authors declare that they have no competing interests.

## Authors’ contributions

'Evangelia Mourmoura conducted the experiments and contributed to the study implementation, statistical analysis, interpretation, and the preparation of the manuscript. Valerie Chaté conducted the *in vivo* experiments and Karine Couturier the OGTT. Brigitte Laillet, Jean-Paul Rigaudière and Béatrice Morio performed the analysis of the fatty acid composition of cardiac phospholipids. Guillaume Vial participated in the animal care. Corinne Malpuech-Brugère and Kasra Azarnoush helped to conduct the experiments and acquire data. Luc Demaison supervised the study conduction and contributed to the study conception and design, implementation, statistical interpretation, the preparation and finalization of the manuscript. All authors approved the final manuscript for publication.
